# Associations between Dietary Acid Load and Biomarkers of Inflammation and Hyperglycemia in Breast Cancer Survivors

**DOI:** 10.3390/nu11081913

**Published:** 2019-08-15

**Authors:** Tianying Wu, Phoebe Seaver, Hector Lemus, Kathryn Hollenbach, Emily Wang, John P. Pierce

**Affiliations:** 1 School of Public Health, Division of Epidemiology and Biostatistics, San Diego State University, San Diego, CA 92182-4162 USA; 2 Moores Cancer Center, University of California at San Diego, San Diego, CA 92093, USA; 3 Department of Pediatrics and Rady Children’s Hospital, University of California at San Diego, San Diego, CA 92093, USA; 4 School of Medicine, Department of Pathology, University of California at San Diego, San Diego, CA 92093, USA

**Keywords:** dietary acid load, inflammation, cancer survivors

## Abstract

Metabolic acidosis can lead to inflammation, tissue damage, and cancer metastasis. Dietary acid load contributes to metabolic acidosis if endogenous acid–base balance is not properly regulated. Breast cancer survivors have reduced capacities to adjust their acid–base balance; yet, the associations between dietary acid load and inflammation and hyperglycemia have not been examined among them. We analyzed data collected from 3042 breast cancer survivors enrolled in the Women’s Healthy Eating and Living (WHEL) Study who had provided detailed dietary intakes and measurements of plasma C-reactive protein (CRP) and hemoglobin A1c (HbA1c). Using a cross-sectional design, we found positive associations between dietary acid load and plasma CRP and HbA1c. In the multivariable-adjusted models, compared to women with the lowest quartile, the intakes of dietary acid load among women with the highest quartile showed 30–33% increases of CRP and 6–9% increases of HbA1c. Our study is the first to demonstrate positive associations between dietary acid load and CRP and HbA1c in breast cancer survivors. Our study identifies a novel dietary factor that may lead to inflammation and hyperglycemia, both of which are strong risk factors for breast cancer recurrence and comorbidities.

## 1. Introduction

Breast cancer is the most common type of cancer in women in the United States [1]. Women who have had breast cancer are at an increased risk of not only cancer recurrence, but also other comorbidities, such as obesity, hypertension, diabetes, dyslipidemia, and decreased bone mass, primarily due to the late effects of cancer treatment and the normal aging processes [2,3]. For instance, Lipscombe et al. demonstrated that post-menopausal breast cancer survivors had higher rates of diabetes than women without breast cancer [4]. Lipscombe et al. followed 24,976 breast cancer survivors and 124,880 age-matched women without breast cancer. After 10 years of follow-up, the risk of diabetes among breast cancer survivors compared to women without breast cancer increased by 20% (HR = 1.21; 95% CI 1.09–1.35) [4].

The measurement of C-reactive protein (CRP) is a marker of general health, longevity, frailty, and overall breast cancer survival. Multiple prospective cohort studies have shown that CRP is a significant predictor for the incidence of cardiovascular disease and other inflammatory-related chronic diseases [5,6]. Emerging evidence also suggests that CRP can predict cancer prognosis and survival in cohort studies [7], and CRP has better predictions than other traditional inflammatory indexes [8]. Hemoglobin A1c (HbA1c) represents long-term blood glucose levels. Elevated HbA1c has been confirmed to be a significant predictor of diabetes, cardiovascular disease, and all-cause mortality as well as breast cancer survival [9,10]. Using these two biomarkers may provide valuable information on the risk of developing comorbidities among women with breast cancer. 

Diet has been found to play an important role in the development of chronic diseases and comorbidities [11]; however, specific dietary guidelines for breast cancer survivors are lacking. Therefore, determining dietary factors in relation to CRP and HbA1c may shed light on whether these dietary factors can influence comorbidities among women with breast cancer. 

The associations between dietary acid load and inflammation and hyperglycemia have not yet been studied in breast cancer survivors. Western diets, consisting of lower fruit and vegetable intake and higher meat consumption, are considered acid-producing diets, whereas a diet with higher fruit and vegetable intake and lower meat and processed grain consumption would be a more alkaline-producing diet [12,13]. Many studies have shown that a diet high in acid load is associated with an increased incidence of chronic kidney disease (CKD), CKD progression [14,15,16], and diabetes [17]. However, the associations between dietary acid load and biomarkers related to inflammation and hyperglycemia have not been studied. Metabolic acidosis can cause tissue damage, which can further initiate inflammation [18,19,20]. Dietary acid load contributes to metabolic acidosis if the acid–base balance is not properly adjusted. Cancer patients have a reduced capacity to adjust the acid–base balance [21]. Thus, dietary acid load may contribute to inflammation in cancer patients. Furthermore, dietary acid load has been shown to increase the risk of type 2 diabetes [17], a disease that manifests signs of hyperglycemia. Hence, dietary acid load may lead to hyperglycemia, especially in breast cancer survivors, who have been shown to have a higher risk of diabetes than women without breast cancer [4]. 

The objective of this study was to conduct a cross-sectional analysis to determine the associations between dietary acid load and CRP and HbA1c, using data from a large cohort study of breast cancer survivors, the Women’s Health Eating and Living (WHEL) study. We hypothesized that there would be a positive association between dietary acid load and biomarkers of HbA1c and CRP. 

## 2. Materials and Methods 

### 2.1. Population

The WHEL Study was a randomized clinical trial conducted between 1995 and 2000 that assigned 3088 breast cancer survivors to either an intensive diet intervention or a comparison group consuming the recommended 5 fruits and vegetables a day; participants were followed through 2006. Detailed selection criteria were previously described [22]. Briefly, inclusion criteria were as follows: Women who had stage I (≥1 cm), II, or IIIA breast cancer diagnosed within the previous 4 years, had no evidence of cancer recurrence, had completed primary therapy, were 18–70 years old at diagnosis, did not have life-threatening comorbidities, and were able to communicate dietary data via 24-h food recall. Exclusions included insulin dependence and the diagnosis of a comorbidity requiring a specific diet or the use of a medication that contraindicated a high-fiber diet and insulin dependence. Women with diagnoses after age 70 years and those with stage 1 tumors smaller than 1 cm were also excluded.

Data on dietary intake, physical activities, lifestyle habits, quality of life and health symptoms, and other medical measures, including types of breast cancer, stage, and types of treatment, were collected at baseline. The WHEL study was approved by the Institutional Review Board (IRB) of the University of California at San Diego. All subjects provided written informed consent. The current study was an ancillary study using the de-identified data from the WHEL study; thus, the exempt IRB was approved by the San Diego State University IRB committee (protocol number: Temp-1286). 

Plasma CRP and HbA1c were measured among all participants using blood samples collected at the comprehensive baseline visits. The current study was conducted using baseline data only; therefore, any dietary intervention from this trial would not affect the dietary information or biomarker levels. Of the original 3088 women who completed the baseline assessment, 46 women were excluded due to missing CRP and HbA1c, leaving 3042 women for analysis. 

### 2.2. Dietary Assessment

Study participants completed four 24-h dietary recalls by telephone on random days over a 3-week period, with two being conducted on the weekends and two on weekdays [22]. Dietary assessors completed a training program and used the multi-pass software-driven recall protocol of the Nutritional Data System software (NDS-R, 1994-2006, University of Minnesota, Minneapolis, MN, USA).

### 2.3. Assessment of Dietary Acid Load

Two scores are commonly used to estimate dietary acid load in epidemiological studies: The Potential Renal Acid Load (PRAL) score and the Net Endogenous Acid Production (NEAP) score. The PRAL score considers the intestinal absorption rates for protein, potassium, calcium, magnesium, and phosphate and has been validated against urine pH in healthy adults [12,23]. A negative PRAL value reflects an alkaline-forming potential, whereas a positive value reflects an acid-forming potential [24]. Frassetto et al. [25] developed the NEAP score, which uses total protein and potassium intake as the main components involved in acid production. The PRAL and NEAP scores were derived from estimations of several nutrient intakes as follows [26]:

PRAL (mEq/day) =  (0.49 × protein (g/day)) + (0.037 × phosphorus (mg/day))  − (0.021 × potassium (mg/day)) − (0.026 × magnesium (mg/day)) − (0.013 × calcium (mg/day))(1)

NEAP (mEq/day) = (54.5 × protein (g/day)/potassium (mEq/day)) − 10.2(2)

This study used both scores for dietary acid assessment because they reflect slightly different nutritional intakes and biological mechanisms.

### 2.4. Measurement of CRP

Serum concentration of CRP was measured via a high-sensitivity electrochemiluminescence assay (MesoScale Discovery, Gaithersburg, MD, USA) by the clinical biochemistry lab at the University of Vermont. The lower detection limit was 0.02 mg/L. The inter-assay coefficients of variation ranged between 7% and 12%. 

### 2.5. Measurement of HbA1c

HbA1c was measured using ion exchange high-performance liquid chromatography (D-10 system; Bio-rad laboratories, Hercules, CA, USA) in washed red blood cells collected at the baseline visit. The coefficients of variation were 1.5% and 1.6%, respectively, for high- and low-quality controls. 

### 2.6. Other Assessments

Demographic characteristics and comorbidities were self-reported. Variables abstracted from patient records included initial cancer diagnosis and treatment. Specific variables abstracted included tumor stage, size, hormone receptor status, and the use of radiation, chemotherapy, and/or post-treatment anti-estrogens. Physical activity levels were assessed using an adapted validated questionnaire from the Women’s Health Initiative. Physical activity was converted into metabolic equivalent tasks (METs) [22]. 

### 2.7. Statistical Analyses

Data analysis was completed using SAS 9.4 (SAS Institute Inc., Cary, NC, USA). We used linear regression models to assess the association between dietary acid load and CRP and HbA1c. CRP and HbA1c were treated as dependent variables, and the biomarker CRP was log transformed because it was not normally distributed. PRAL and NEAP scores were categorized into quartiles and treated as independent variables. PRAL and NEAP scores were entered into the linear regression model separately, not simultaneously, because these two variables are highly correlated. We assessed potential confounders based on their correlations with dietary acid load and CRP or HbA1c. We also assessed potential confounders based on information in previous literature. In multivariable models, we adjusted for the following confounders based on a priori assumption: Age at diagnosis (continuous), ethnicity (White, Black, Hispanic, Other/Mixed race), physical activities (METS at 0–600, 600–1200, >1200 min/week), body mass index (normal, overweight, obese), smoking status (current, former, never), caloric intake (continuous), menopausal status (premenopausal, postmenopausal, perimenopausal), and cancer characteristics—namely, tumor stage (I, II, IIIA) and size (continuous), hormonal receptor status (ER+/PR+, ER+/PR-, ER-/PR+, ER-/PR-), radiation (yes, no), and chemotherapy (yes, no). Additional covariates related to use of medicines were adjusted during the analyses. Because these covariates did not change the final estimates, they were removed from the final model. These covariates included use of anti-estrogen therapies (i.e., tamoxifen or other anti-estrogens) and use of other medications (i.e., cardiovascular medicines, blood-sugar controlling medicines, corticosteroids, and gastrointestinal medicines), as presented in Table 1. 

Stratified analyses were conducted to determine the association between dietary acid load and biomarkers based on pack-years of smoking with no current smokers included (0 vs. >0). To assess whether there was significant interaction between dietary acid load and any subgroup strata, we used the Wald *p*-value for the interaction term in a model that also included the main effects. 

To determine whether each individual component in the PRAL formula had an independent association with a biomarker, we controlled total protein (or animal or vegetable protein), phosphorus, calcium, magnesium, and potassium as the independent variables in the multivariable regression models. In addition, to validate the associations of PRAL with a biomarker, we assessed the associations of PRAL-contributing foods with this biomarker. We further mutually adjusted PRAL together with PRAL-contributing foods to determine whether additional components (other than components in PRAL) in these foods made the contributions. 

## 3. Results

Baseline characteristics of the overall 3042 women are displayed in Table 1. The majority of patients were white (*n* = 2588), postmenopausal women (*n* = 2399), overweight or obese (*n* = 1674), and 50 years old at diagnosis (mean = 50.74 ± 8.85). Approximately 95% had stage I or II tumors (*n* = 2827) and 63% had estrogen receptor- and progesterone receptor-positive (ER+/PR+) tumors (*n* = 1908).

Table 2 shows the descriptive statistics by quartile of PRAL. Compared to women with a lower dietary acid load, women with higher PRAL tended to be younger, obese, and premenopausal women, less physically active, and more likely to receive chemotherapy. 

As shown in Table 3, both age- and multivariable-adjusted analyses demonstrated significant associations. In the multivariable-adjusted analyses, women at the highest quartile of PRAL had 33% higher CRP levels (*p*-value < 0.0001) than those in the lowest quartile of PRAL, while the average HbA1c levels were 0.091 (*p*-value = 0.0098) higher in the highest quartile of PRAL than the lowest quartile of PRAL. Similar magnitudes and significances of the associations were observed for NEAP. In multivariable-adjusted analyses, *p*-values for trends were significant for both PRAL and NEAP for the CRP biomarker (<0.001 for all) and for PRAL for the HbA1c biomarker, but were only marginally significant for NEAP for the HbA1c biomarker.

Table 4 presents the stratified analyses determining the association between dietary acid and CRP according to pack-year of smoking: Pack-year = 0 and >0. Current smokers were removed from the stratified analyses. In the strata of pack-years > 0, women in the highest quartile of NEAP had 39.5% higher levels of CRP (*p*-value < 0.0001) than women in the lowest quartile. Comparatively, in women with zero pack-years of smoking, women in the highest quartile of NEAP had 25.8% higher levels of CRP (*p*-value < 0.002) than those in the lowest quartile. Similar trends were observed for the associations between PRAL and CRP in the two pack-year strata. There were marginal interactions between pack-years and dietary acid load: The *p*-values for interactions were 0.06 for NEAP and 0.13 for PRAL. 

In addition, we examined whether each component in PRAL was associated with CRP (see Supplementary Table S1). We found that total protein was positively associated and potassium and magnesium were negatively associated with CRP in model 1 (*p* for trend < 0.05 for all three). In model 2, we further separated total protein into animal and vegetable protein. We observed opposite trends of animal and vegetable protein, although vegetable protein was only marginally significant. Magnesium was no longer significant in model 2. Moreover, we determined the associations of PRAL-contributing foods with CRP (Supplementary Table S2). Fresh and processed red meat were acid-producing foods, whereas different types of vegetables were alkaline-producing foods. Compared to tertile 1, beta estimates for tertile 3 were −0.11 for cruciferous vegetables (*p*-value = 0.02), −0.09 for soy legumes (*p*-value = 0.05), 0.13 for fresh red meat (*p*-value = 0.03), and 0.10 for processed red meat (*p*-value = 0.06). The four estimates showed *p* for trend ≤ 0.05. These associations remained significant or marginally significant after adjustment of PRAL. We found that PRAL was still significantly and positively associated with CRP, even after controlling several PRAL-contributing foods (beta was 0.26 between extreme quartiles, *p* < 0.001; *p* for trend < 0.001).

## 4. Discussion

Our study is the first study investigating the associations between dietary acid load and inflammation and hyperglycemia in breast cancer survivors. The results indicated that a higher dietary acid load, characterized by a higher PRAL or NEAP score, was associated with an increase of CRP and HbA1c. Associations were linear and tended to be stronger for CRP as well as similar across the two dietary acid scores. We further found that positive associations between dietary acid and CRP were stronger in past smokers with higher pack-years (>0). 

The positive associations between dietary acid load and HbA1c support previous studies [17,27,28]. Increased dietary acid load was significantly associated with an increased risk of diabetes and insulin resistance [17,27,28]. However, these studies were conducted among healthy individuals; ours was the first study among breast cancer women, who have been shown to have an increased risk of diabetes compared to women without breast cancer [4].

Although few epidemiologic studies have examined the associations between dietary acid load and systemic inflammation in breast cancer survivors or healthy individuals, some animal studies may help explain our findings. One biological explanation for the relationship between dietary acid load and inflammation is that metabolic acidosis can cause tissue damage, which can further initiate inflammatory responses [29]. Inflammation is the body’s natural response to injury. It works to heal wounds, such as damage to tissue or blood vessels. Animal studies have shown that acidosis caused lung and intestinal damage, stimulated the expression of inflammatory signaling molecules such as induced nitric oxide synthases, increased activities of inflammatory enzymes such as myeloperoxidase, and increased levels of inflammatory cytokines such as tumor necrosis factor (TNF) [18,19,20]. Chronic inflammation can certainly play a role in cancer progression [30,31]. In previous studies, patients with higher CRP levels at the time of the breast cancer diagnosis had increased breast cancer recurrence [32] and a reduction in both overall and disease-free survival time [33]. Elevated CRP levels have also been found in other chronic diseases, such as diabetes [34]. The positive relationship between dietary acid load and inflammation is particularly important in breast cancer survivors, as they are at higher risk of cancer recurrence and inflammation-associated comorbidities, such as diabetes and cardiovascular disease [2,4]. Therefore, dietary acid load could potentially provide patients with modifiable dietary targets for reducing their comorbidities and improving their quality of life.

We found marginal interactions between pack-year and dietary acid load. The association between dietary acid load and CRP was stronger in women with pack-years of smoking >0 than those with pack-years of smoking equal to 0. Pack-years can better indicate the intensity of smoking in former smokers. One pack-year is equal to smoking one pack per day for one year or two packs per day for half a year. In the WHEL cohort, 41% were past smokers while only 4.5% were current smokers. Because current smoking can significantly elevate CRP levels [35,36], we removed current smokers from our dataset to tease out the impact of dietary acid load on inflammation. Acid–base balance is usually adjusted by electrolytes and the respiratory system, then by the kidneys and bones; however, smoking can significantly disturb electrolytes, damage lung and kidney function, and reduce bone mineral density [37,38,39,40,41]. Therefore, past smokers with higher smoking intensity will have a lower capacity to regulate their acid–base balance than those who never smoked, as quitting smoking cannot remove past damages. The results of our study demonstrate differential responses to dietary acid load across smoking status and provide more evidence for individualized nutrition. 

Additional analyses in two supplementary tables further confirmed that components in PRAL better predicted CRP when they were analyzed jointly, using the PRAL formula rather than analyzed individually. Only a few components remained significant when they were analyzed individually. Magnesium was no longer significant after the adjustment of vegetable protein, suggesting that other components—but not magnesium—in vegetable proteins were associated with CRP. Vegetable protein is a rich source of magnesium [42]; however, plants enriched with vegetable protein may also have other components, such as isoflavones in soy legumes, which may protect against inflammation [43]. The inverse association between soy legumes and CRP presented in Supplementary Table S2 further supports the plausibility of this mechanism. Furthermore, results on PRAL-contributing foods were in line with PRAL results. We found some acid-producing foods were positively associated and some alkaline-producing foods were inversely associated with CRP. Moreover, these foods remained significantly associated with CRP even after controlling PRAL, indicating that other components in these foods may also play a role in these associations. For instance, red meat has not only protein and phosphorus, but also a sugar-like molecule, N-glycolylneuraminic acid, which can promote inflammation [44]. Similarly, in addition to being an alkaline-contributing food, cruciferous vegetables are a primary dietary source of isothiocyanates and indoleschemicals, which have been shown to have anti-inflammatory functions [45,46]. PRAL was independently associated with CRP even after controlling PRAL-contributing foods, indicating that PRAL better predicted the associations of dietary acid load with CRP than some PRAL-contributing foods. Identifying active components in food and determining whether each component influences biomarkers independently or jointly will help create efficient strategies for chronic disease prevention [47].

### Strengths and Limitations

There are several strengths of this study. Our study is the first study that determines the association between dietary acid load and inflammation and hyperglycemia in a population of breast cancer survivors. We utilized a large existing cohort of breast cancer survivors, which enabled us to account for multiple confounders in multivariable-adjusted models. The WHEL cohort provides more detailed and accurate usual dietary intakes of participants’ diets because the 24-h recalls were collected four times at baseline. Many large cohorts focusing on breast cancer survivors collect only food frequency questionnaires, not multiple 24-h recalls for every individual. However, there are several limitations of this study. This study is a post hoc analysis of large clinical trial data from the WHEL study; therefore a causal-effect conclusion cannot be obtained. Because of the cross-sectional nature, we cannot make causal inferences. Longitudinal studies are warranted to determine the effect of dietary acid load on CRP and HbA1c. There is also the possibility of volunteer bias, as the WHEL cohort was a voluntary intervention study. The women in the WHEL cohort were highly motivated, health-conscious breast cancer survivors. This cohort was comprised of primarily white women with breast cancer; thus, the results of our study may not be generalizable to other ethnic groups of breast cancer survivors, or to women without breast cancer. Although adjustment of hormone receptor statuses (ER/PR statuses) did not change the estimates in the multivariable models, HER2 status was not collected in the WHEL cohort, which may have resulted in residual confounding. Finally, our results could be confounded by medications. To reduce confounding by medications, we adjusted chemotherapy, anti-estrogen therapy, use of corticosteroids, and other medications used for cardiovascular diseases, blood sugar disorders, and gastrointestinal issues. The adjustment of these treatments did not materially change these associations, which can further assure us that our findings were not at least confounded by these medications. Nevertheless, our questionnaires related to medications may not be detailed enough to catch all the medications; thus, residual confounding cannot be ruled out. 

## 5. Conclusions

The positive associations between dietary acid load and CRP and HbA1c have important implications for breast cancer survivors. For example, decreasing dietary acid load can be recommended as a prevention strategy to reduce inflammation and hyperglycemia. This would include a diet that is more alkaline-producing, such as one containing nuts, fruits, and vegetables, but less meat, processed grain, and dairy products. This recommendation is particularly important for breast cancer survivors who are former smokers and have a higher intensity of smoking history because these women have a reduced capacity to regulate the acid–base balance and are already at a higher risk of recurrence and comorbidities.

## Figures and Tables

**Table 1:** Baseline characteristics of breast cancer survivors in the Women’s Health Eating and Living (WHEL) cohort (*n* = 3042).

Characteristics	All Participants
**Age at diagnosis, Mean (SD), Year**	50.7 (8.9)
**Ethnicity, N (%)**	2551 (85.7)
White	
African American	105 (3.5)
Hispanic	159 (5.3)
**BMI, N (%)**	1274 (42.8)
Normal	
Overweight	928 (31.2)
Obese	775 (26.0)
**Smoking Status, N (%)**	132 (4.4)
Current	
Former	1230 (41.3)
Never	1585 (53.2)
**Menopause Status, N (%)**	332 (11.2)
Premenopausal	
Postmenopausal	2370 (79.6)
Perimenopausal	270 (9.1)
**Stage, N (%)**	1153 (38.7)
I	
II	1674 (56.2)
IIIA	150 (5.0)
**Hormone receptor, N (%)** ER+/PR+	1908 (63.1)
ER-/PR+	360 (12.1)
ER+/PR-	128 (4.3)
ER-/PR-	607 (20.4)
**Radiation, N (%)**	1134 (38.1)
No	
Yes	1840 (61.8)
**Chemotherapy, N (%)**	903 (30.3)
No	
Yes	2073 (69.6)
**Anti-estrogen therapy, N (%)**	
Tamoxifen	2009 (66.0)
Other anti-estrogens	72 (2.4)
None or unknown	961 (31.6)
**Other medications, N (%)**	
Cardiovascular medicine	383 (12.6)
Blood sugar medicine or	45 (1.5)
corticosteroids	
Gastrointestinal medicine	206(6.8)
None of the above	2408 (79.0)
**METS minutes/week, N (%)**	1149 (38.6)
0–600	
600–1200	702 (23.6)
>1200	807 (27.1)

BMI denotes body mass index; METS denotes exercise metabolic equivalents in minutes per week; SD denotes standard deviation.

**Table 2:** Descriptive statistics of breast cancer survivors in the WHEL cohort by quartile of Potential Renal Acid Load (PRAL) (*n* = 3042).

		PRAL		
	1	2	3	4
**Age at diagnosis, mean (SD)**	52.6 (8.3)	51.7 (8.8)	50.5 (8.8)	48.5 (9.0)
**Ethnicity, N (%)**				
White	682 (89.7)	677 (89.3)	635 (84.6)	587 (78.6)
African American	7 (0.9)	16 (2.1)	20 (2.7)	65 (8.7)
Hispanic	26 (3.4)	35 (4.6)	45 (6.0)	55 (7.3)
Other/Mixed Race	45 (5.9)	30 (4.0)	51 (6.8)	40 (5.4)
**BMI, N (%)**	429 (56.4)	355 (46.8)	275 (36.6)	243 (32.5)
Normal				
Overweight	223 (29.3)	230 (30.3)	268 (35.7)	211 (28.2)
Obese	108 (14.2)	173 (22.8)	208 (27.4)	293 (39.2)
**Smoking Status, N (%)**	24 (3.2)	34 (4.5)	37 (5.0)	40 (5.4)
Current				
Former	324 (43.1)	306 (40.6)	314 (42.3)	303 (41.0)
Never	403 (53.7)	413 (54.8)	392 (52.8)	396 (53.6)
**METS minutes/week, N (%)**	230 (32.6)	286 (41.6)	313 (47.2)	328 (51.7)
0–600				
600–1200	206 (29.2)	184 (26.8)	185 (27.9)	139 (21.9)
>1200	269 (38.2)	217 (31.6)	165 (24.8)	168 (26.5)
**Menopause Status, N (%)**	59 (7.8)	74 (9.8)	89 (11.9)	116 (15.5)
Premenopausal				
Postmenopausal	640 (84.3)	609 (80.7)	599 (79.8)	546 (73.2)
Perimenopausal	60 (7.9)	72 (9.5)	63 (8.4)	84 (11.3)
**Stage, N (%)**	298 (39.2)	283 (37.3)	289 (38.5)	295 (39.5)
I				
II	418 (55.0)	448 (59.1)	427 (56.9)	406 (54.4)
IIIA	44 (5.8)	27 (3.6)	35 (4.7)	46 (6.2)
**Hormone Receptor Status, N (%)**	488 (64.2)	489 (64.5)	480 (63.9)	448 (60.0)
ER+/PR+				
ER-/PR+	115 (15.1)	92 (12.1)	72 (9.6)	86 (11.5)
ER+/PR-	30 (3.9)	34 (4.5)	30 (4.0)	34 (4.6)
ER-/PR-	127 (16.7)	143 (18.9)	169 (22.5)	179 (24.0)
**Radiation, N (%)**	275 (36.2)	292 (38.5)	305 (40.6)	276 (37.0)
No				
Yes	485 (63.8)	466 (61.5)	444 (59.1)	470 (62.9)
**Chemotherapy, N (%)**	261 (34.3)	237 (31.3)	212 (28.2)	199 (26.6)
No				
Yes	499 (65.7)	521 (68.7)	538 (71.6)	548 (73.4)

BMI denotes body mass index; METS denotes metabolic equivalents in minutes per week; SD denotes standard deviation.

**Table 3:** Age-adjusted and multivariable-adjusted associations between dietary acid load (PRAL and Net Endogenous Acid Production (NEAP)) and biomarkers (C-reactive protein (CRP) and hemoglobin A1c (HbA1c)).

		**PRAL**				
		**Quartile 1**	**Quartile 2**	**Quartile 3**	**Quartile 4**	
	Median	−21.046	−8.28	0.09	11.02	
			**Beta (*p*-value)**	**Beta (*p*-value)**	**Beta (*p*-value)**	***P* for trend**
**CRP**	Age-adjusted	Ref	0.18 (<0.0001)	0.54 (<0.0001)	0.84 (<0.0001)	<0.0001
	Multivariable-adjusted	Ref	0.09 (0.001)	0.22 (0.0001)	0.33 (<0.0001)	<0.0001
**HbA1c**	Age-adjusted	Ref	0.05 (0.002)	0.14 (<0.0001)	0.21 (<0.0001)	<0.0001
	Multivariable-adjusted	Ref	0.04 (0.03)	0.07 (0.04)	0.09 (0.01)	0.01
		**NEAP**				
		**Quartile 1**	**Quartile 2**	**Quartile 3**	**Quartile 4**	
	Median	23.01	33.13	41.74	54.82	
			**Beta (*p*-value)**	**Beta (*p*-value)**	**Beta (*p*-value)**	***P* for trend**
**CRP**	Age-adjusted	Ref	0.16 (<0.0001)	0.56 (<0.0001)	0.83 (<0.0001)	<0.0001
	Multivariable-adjusted	Ref	0.08 (0.0072)	0.23 (<0.0001)	0.31 (<0.001)	<0.0001
**HbA1c**	Age-adjusted	Ref	0.041 (0.01)	0.14 (<0.0001)	0.19 (<0.0001)	<0.0001
	Multivariable-adjusted	Ref	0.03 (0.13)	0.06 (0.07)	0.06 (0.08)	0.10

Multivariable models were adjusted for ethnicity, age at diagnosis, physical activity, body mass index, smoking status, total energy intakes, menopausal status, and cancer characteristics—namely, tumor stage, tumor size, hormonal status, radiation, and chemotherapy. PRAL denotes potential renal acid load; NEAP denotes net endogenous acid production; CRP denotes C-reactive protein; HbA1c denotes hemoglobin A1c.

**Table 4:** Multivariable-adjusted associations between dietary acid load (PRAL and NEAP) and CRP, stratified by pack-years of smoking.

**CRP**		**PRAL**				
		**Q1**	**Q2**	**Q3**	**Q4**	
	**n**	**Ref**	**Beta (*p*-value)**	**Beta (*p*-value)**	**Beta (*p*-value)**	***P* for Interaction**
**Pack-Years**						0.14
0	1643		0.11 (0.003)	0.23 (0.004)	0.22 (0.007)	
>0	1256		0.04 (0.31)	0.23 (0.01)	0.44 (<0.0001)	
**CRP**		**NEAP**				
		**Q1**	**Q2**	**Q3**	**Q4**	
	***n***	**Ref**	**Beta (*p*-value)**	**Beta (*p*-value)**	**Beta (*p*-value)**	***P* for Interaction**
**Pack-Years**						0.06
0	1643		0.08 (0.05)	0.26 (0.001)	0.26 (0.002)	
>0	1256		0.09 (0.04)	0.22 (0.01)	0.40 (< 0.0001)	

Multivariable models were adjusted for ethnicity, age at diagnosis, physical activity, body mass index, smoking status, total energy intakes, menopausal status, and cancer characteristics—namely, tumor stage, tumor size, hormonal status, radiation, and chemotherapy. *P*-values for trend showed significant increasing trends across all quartiles of dietary acid load. PRAL denotes potential renal acid load; NEAP denotes net endogenous acid production; CRP denotes C-reactive protein; HbA1c denotes hemoglobin A1c.

## Supplementary Materials

The following are available online at https://www.mdpi.com/2072-6643/11/8/1913/s1, Table S1: The associations between PRAL components and CRP, Table S2: The associations of PRAL and PRAL-contributing foods with CRP.
